# Multi-omics and network pharmacology reveal Huayu-Tongbi decoction reduced arthritis-related bone erosion

**DOI:** 10.1186/s13020-025-01159-1

**Published:** 2025-07-02

**Authors:** Bozhen Chen, Lu Yang, Houchun Wang, Peng Yu, Mengyang Ma, Meiqi Chen, Yingyan Zhou, Jiaqi Wu, Huasheng Liang, Maojie Wang, Runyue Huang, Yiting He, Qingchun Huang, Xiaohong He

**Affiliations:** 1 Second Clinical Medical College of Guangzhou University of Chinese Medicine, No.111, Dade Road, Yuexiu District, Guangzhou City, 510120 Guangdong People’s Republic of China; 2 Guangdong-Hong Kong-Macau Joint Lab on Chinese Medicine and Immune Disease Research, Guangzhou University of Chinese Medicine, Guangzhou, China; 3 Guangdong Provincial Key Laboratory of Clinical Research on Traditional Chinese Medicine Syndrome, Guangzhou, China; 4 Luoyang Orthopedic Hospital of Henan Province & Orthopedic Hospital of Henan Province, Luoyang, Henan China; 5 Guangdong Provincial Key Laboratory of Chinese Medicine for Prevention and Treatment of Refractory Chronic Diseases, Guangzhou, China; 6 State Key Laboratory of Dampness Syndrome of Chinese Medicine, The Second Affiliated Hospital of Guangzhou University of Chinese Medicine (Guangdong Provincial Hospital of Chinese Medicine), Guangzhou, China; 7 The Second Affiliated Hospital, Guangzhou University of Chinese Medicine (Guangdong Provincial Hospital of Chinese Medicine), Guangzhou, China

**Keywords:** Traditional Chinese Medicine, Rheumatoid arthritis, Bone erosion, Huayu-Tongbi decoction, Multiomics, Network pharmacology

## Abstract

**Background:**

Rheumatoid arthritis (RA), an autoimmune disorder marked by joint inflammation and bone destruction, lacks effective therapies targeting bone erosion. Huayu-Tongbi decoction (HT), a traditional Chinese medicine (TCM) herbal decoction, has been used as a complementary treatment for RA, yet the mechanisms of its active components and multitarget therapeutic effects remain unclear.

**Materials and methods:**

An adjuvant-induced arthritis (AIA) model was established in rats, and enzyme-linked immunosorbent assay, histopathological staining, and micro–Computed Tomography to assess the effects of HT on joint inflammation and bone erosion. Furthermore, serum pharmacochemistry combined with network pharmacology identified the HT’s active ingredients and targets. In vitro multi-omics study revealed the decoction’s effect and underlying mechanisms in osteoclastic differentiation.

**Results:**

HT significantly reduced joint inflammation and bone erosion in AIA rats. Serum pharmacochemistry identified 44 absorbed components in HT, and network pharmacology analysis predicted 89 key targets of HT related to RA. In vitro experiments demonstrated that HT inhibited RANKL–induced osteoclastic differentiation through multiple pathways, such as PPAR pathway, AA metabolism, and NF-κB pathway.

**Conclusion:**

This study confirmed the beneficial effects of HT in experimental arthritis and explored the specific mechanisms involved. HT inhibited osteoclastic differentiation through multiple targets and pathways to reduced bone destructions, providing a potential therapeutic strategy for preventing RA-related bone erosion.

**Graphical abstract:**

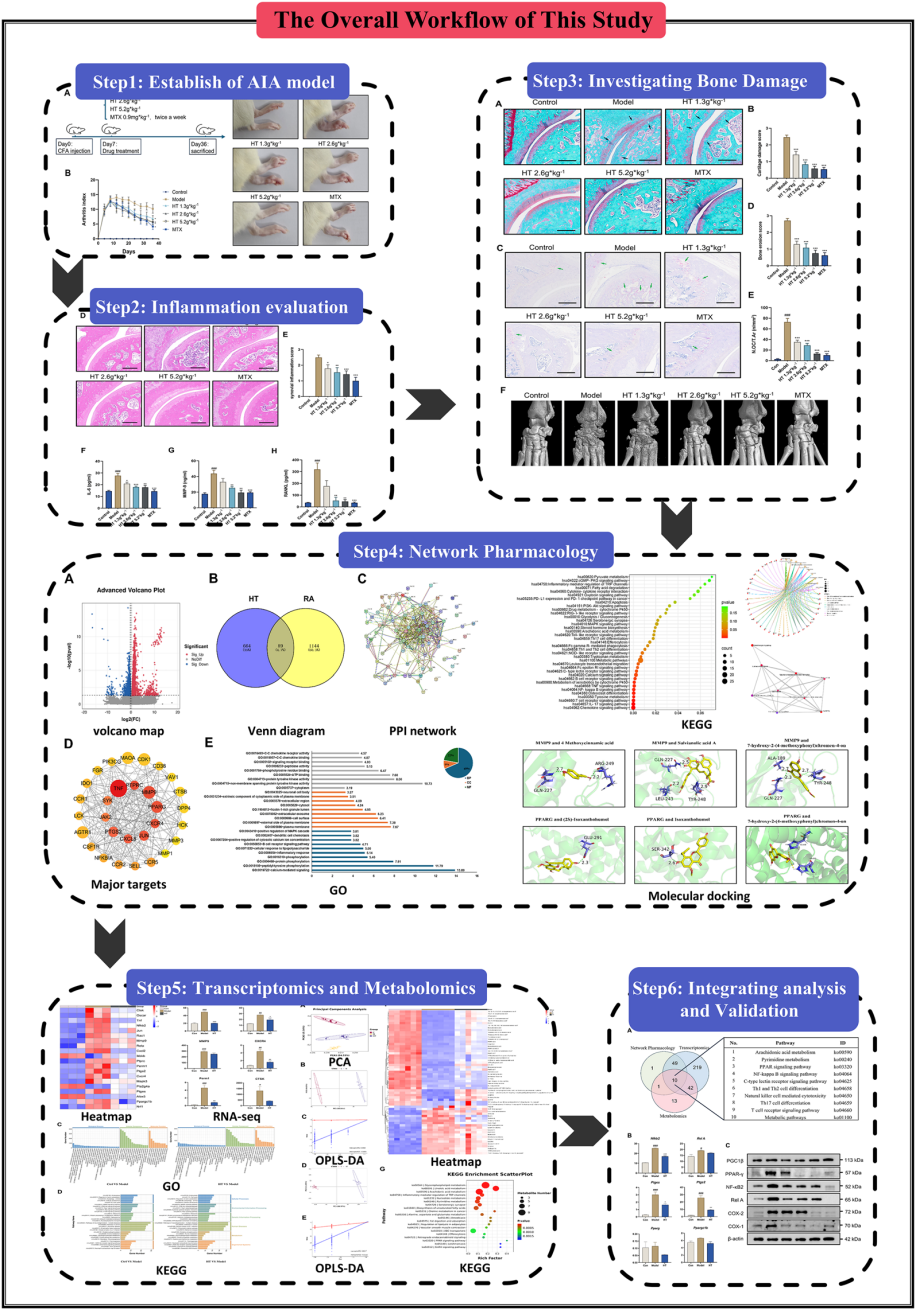

**Supplementary Information:**

The online version contains supplementary material available at 10.1186/s13020-025-01159-1.

## Background

Rheumatoid arthritis (RA) is a common autoimmune disease, affecting more than 1% of the world population; it reduces patients’ mobility and quality of life [1]. The main features of RA are joint inflammation and bone erosion [2]; the latter, presents as localized degradation of bone tissue within inflamed joint, periarticular around the sites of inflammation. Progressive articular tissue deterioration results in irreversible joint damage and significant functional impairment. These resorptive processes are principally mediated by osteoclasts (OCs), which are the exclusive mediators of physiological and pathological bone resorption. OCs constitute the final common pathway for all types of RA-related bone erosion. Current therapies for RA, like Methotrexate (MTX), are not effective against bone erosion and are associated with adverse effects such as myelosuppression; therefore, developing new therapies that specifically target joint-related pathogenic molecules or cells remains a critical need.

Huayu-Tongbi decoction (HT), a Chinese herbal decoction, has been used as a complementary traditional medicine in RA treatment. It was developed by Prof. Qingchun Huang through optimizing *Wutou Tang* (Aconite Decoction) from *Jin Gui Yao Lüe* (*Synopsis of the Golden Chamber*), with the addition of *ChuanShanLong* (*Dioscorea nipponica subsp. Rosthornii* (Diels) C.T.Ting). *ChuanShanLong* is a medicinal plant traditionally used in Chinese folk medicine to treat arthritis and has been widely documented in regional herbal compendiums, such as *Dongbei Yaozhi Zhi* (*Northeast China Medicinal Plants Compendium*) and *Shaanxi Zhongcaoyao* (*Shaanxi Chinese Herbal Medicine*). This herbal prescription has demonstrated positive outcomes in clinical studies as a complementary RA treatment that alleviates RA-related symptoms [3]. HT suppresses inflammatory proliferation of fibroblast-like synoviocytes through the nuclear factor κ-light-chain-enhancer of activated B cells (NF-κB) and Janus-associated kinase/signal transducer and activator of transcription (JAK/STAT) signaling pathways [4]. However, the mechanisms and active components contributing to HT as a multi-target treatment, and the decoction’s influence on bone erosion in inflammatory arthritis, are still unknown.

Traditional Chinese medicine (TCM) is renowned for its holistic, multi-target therapeutic effects. Due to the complexity of TCM components, analyzing drug constituents and identifying active compounds are necessary for studying the efficacy of such medications [5, 6]. Therefore, in this study, we chose the emerging strategy of network pharmacology analysis combined with multiomics to discover the active ingredients and targets of HT. This approach enabled systematic prediction of ingredient–target interactions while compensating for single-method limitations via cross-validation, thereby establishing comprehensive “component–target–pathway” networks that elucidated therapeutic mechanisms at both the molecular and systemic levels.

In this study, we systematically evaluated HT anti-arthritic efficacy using an experimental arthritis model, utilizing serum pharmacochemistry combined with network pharmacology to predict the active ingredients and targets of HT. Notably, HT not only suppressed joint inflammation, but also reduced related bone erosion, shows the potential directly bone protective effect. To confirming the predicted mechanisms. Multi-omics were applied to further evaluate the mechanism of the drug-containing serum in vitro*,* confirming that HT reduced osteoclast differentiation, and evaluate its mechanism.

## Materials and methods

### Reagents

#### Chemical reagents

Standardized herbal decoction pieces were purchased from Kangmei Pharmaceutical Co., Ltd. (Guangdong, China). We bought complete Freund’s adjuvant (CFA) containing 10 mg/mL heat-killed mycobacteria (#7027) from Chondrex (Redmond, WA, USA). MTX was obtained from Shanghai Sine Pharmaceutical Laboratories Co., Ltd. (Shanghai, China). We purchased interleukin-6 (IL-6), receptor activator of nuclear factor κ-β ligand (RANKL), and matrix metalloproteinase-9 (MMP-9) enzyme-linked immunosorbent assay (ELISA) kits from Cusabio Biotech (Wuhan, China). A hematoxylin and eosin (H&E) staining kit was obtained from Wuhan Servicebio Technology Co., Ltd. (Wuhan, China). We purchased a Tartrate-resistant Acid Phosphatase (TARP) Staining Kit (#294-67001) from Wako Pure Chemical Industries, Ltd. (Tokyo, Japan). A Safranin O/Fast Green Staining Kit was bought from Pinuofei Biological Technology Co., Ltd. (Wuhan, China). We obtained a Cell Counting Kit-8 (CCK-8; #C0089) from Beyotime Institute of Biotechnology Co. (Shanghai, China). TRIzol reagent and was purchased from Thermo Fisher Scientific, Inc. (Waltham, MA, USA). RANKL (#HY-P7425) and M-CSF (#HY-P7085) was purchased from MedChemExpress LLC (MCE, Shanghai, China). Zoledronic acid (Zol, #1724827 was purchased from Merck KGaA (Darmstadt, Germany). Primary antibody [COX2(cst#12282s), COX1(cst#9896), NFκB2 (cst#3017p), Rel A(cst#6956), β-actin(cst#8457)] were purchased from Cell Signaling Technology, Inc (Boston, USA) and PGC1β(ab176328) were purchased from Abcam Limited. (Cambridge, UK) and PPAR-y(sc7273) were purchased from Santa Cruz Biotechnology, Inc. (Texas, USA).

#### Preparation of HT

HT is composed of seven herbs that have been used in China for centuries. Table 1 lists the components of these herbs. First, we boiled Radix Aconiti Carmichaeli (previously pre-cooked and sliced) in 200 mL water for 30 min to decrease its inherent toxicity. The remaining herbal mixture, which had been pre-soaked in water for 30 min, was added and further boiled in water at 8:1 (v/w) for 1 h. We collected the extracted liquid, added the herb residue to deionized water, and boiled this mixture for another 30 min. The liquid extracted from this process was collected and combined with the earlier extraction. After concentrating this mixture (decoction piece weight/body weight [BW]) using a rotary evaporator (#R502B; SENCO Technology, Shanghai, China); we stored it at 4°C and used it within 1 week.

**Table 1:** The components of HT

Components	Chinese name	Scientific name	WFO ID	Weight
Rhizoma Dioscoreae Nipponicae	ChuanShanLong	*Dioscorea nipponica subsp. rosthornii *(Diels) C.T.Ting	wfo-0000392874	30 g
Salvia miltiorrhiza	DanShen	*Salvia miltiorrhiza* Bunge	wfo-0000301608	20 g
Astragalus Mongholicus	HuangQi	*Astragalus mongholicus* Bunge	wfo-0000185128	20 g
Radix Paeoniae Alba	BaiShao	*Paeonia lactiflora* Pall	wfo-0000480479	20 g
Rhizoma Corydalis	YanHuSuo	*Corydalis turtschaninovii f. yanhusuo* Y.H.Chou & Chun C.Hsu	wfo-0000623011	10 g
Radix Aconiti Carmichaeli (Cooked and sliced)	FuZi	*Aconitum carmichaelii* Debeaux	wfo-0000517046	10 g
Radix Glycyrrhizae	GanCao	*Glycyrrhiza uralensis* Fisch. ex DC	wfo-0001053609	6 g

### In vivo experiment

#### Animal administration

We established an adjuvant-induced arthritis (AIA) model in healthy male Wistar rats (200 ± 20 g). After acclimatization feeding, rats were subcutaneously injected with 100 μl CFA containing 10 mg/mL heat-killed mycobacteria or normal saline as control at the base of their tails. Eight days after disease induction, The HT group rats received daily oral gavage administration of HT in different concentrations (1.3 g × kg^−1^, 2.6 g × kg^−1^, and 5.2 g × kg^−1^, equivalent to 1/2, 1, and 2 times the clinical equivalent dose, respectively) or received the same volume of normal saline as normal control. For positive control, rats received MTX solution (0.9 mg/kg) to at a dosage of 1 mL/100 g intragastrically, with equivalent-volume saline solution administered on non-treatment day. After 32 days of treatment, rats in both groups were kept fasting and water-free for 12 h and inducted of anesthesia with 5% isoflurane, and then sacrificed by cervical dislocation under deep anesthesia.

All animal administration were approved by the Experimental Animal Research Center of the Second Affiliated Hospital of GuangZhou University of Chinese Medicine (GZUCM, Permit No. 2022107) and supervised by the Institutional Animal Care and Use Committee of GZUCM.

#### Enzyme-linked immunosorbent assay (ELISA)

The levels of IL-6, RANKL and MMP-9 in the serum were measured by ELISA according to the manufacturer’s instructions.

#### Micro–computed tomography (micro-CT)

After fixing joint tissues in 4% paraformaldehyde, samples were then placed in a Skyscan 1276 micro-CT Scanner (Bruker [UK] Ltd., Coventry, UK) and scanned at 60 kV and 50 W to obtain two-dimensional projection images. Subsequently, we reconstructed the 3D images using CTVox software and analyzed them using CT-Analyser (CTAn) software (both Bruker).

#### Histopathology

##### Hematoxylin and eosin staining

The H&E staining was performed according to the manufacturer’s protocol.

##### Tartrate-resistant acid phosphatase staining

At the study endpoint, we stained samples using the TRAP Staining Kit, scanned them on a VS200 Slide Scanner (OLYMPUS, Tokyo, Japan), and quantified TRAP-stained polynucleated OCs (≥ 3 nuclei). The number and area of OCs were measured using ImageJ software (National Institutes of Health [NIH], Bethesda, MD, USA).

##### Safranin O/Fast Green staining

Staining was performed following the instructions on the product datasheet. We evaluated levels of cartilage damage and bone erosion following the recommendations for Standardised Microscopic Arthritis Scoring of Histological sections [7].

### Serum pharmacochemistry and network analysis

#### Preparation of HT-containing serum

We randomly divided male Sprague Dawley rats into two groups, control and HT (n = 15 per group). The HT group was administered 5.2 g × kg^ − 1^ HT intragastrically at a dosage of 1 mL/100 g daily for 7 days; the control group received the same volume of water. Sixty minutes after the final administration, rats were inducted of anesthesia with 5% isoflurane. we obtained blood samples from the rats and centrifuged them at 3500 rpm for 10 min. Germ-free sera containing drug metabolites were prepared under sterile conditions and then stored at − 80 °C.

#### Liquid chromatography with tandem mass-spectrometry analyses (LC–MS/MS) for serum pharmacochemistry

Analysis of HT and its absorbed components in rat serum, sample preparation, liquid chromatography with tandem mass spectrometry (LC–MS/MS), and data pre-processing were performed as previously reported [8]. Briefly, LC–MS/MS analyses were performed using an UHPLC system (Vanquish, Thermo-Fisher Scientific) with a Phenomenex Kinetex C18 (2.1 mm*100 mm, 2.6 μm) coupled to Orbitrap Exploris 120 mass spectrometer (Orbitrap MS, Thermo). The mobile phase A:0.01% acetic acid in water; mobile phase B: IPA: ACN (1:1, v/v). The auto-sampler temperature was 4 °C, and the injection volume was 2 μL. The Orbitrap Exploris 120 mass spectrometer was used for its ability to acquire MS/MS spectra on information-dependent acquisition (IDA) mode in the control of the acquisition software (Xcalibur, Thermo). In this mode, the acquisition software continuously evaluates the full scan MS spectrum. The ESI source conditions were set as following: sheath gas flow rate as 50 Arb, Aux gas flow rate as 15 Arb, capillary temperature 320 °C, full MS resolution as 60000, MS/MS resolution as 15000, collision energy: SNCE 20/30/40, spray voltage as 3.8 kV (positive) or -3.4 kV (negative), respectively. After converting the raw data into mzXML format using the ProteoWizard software, metabolite identification was performed using the Biotree TCM (V 1.0) database and BT-HERB (V 1.0) and visualized.

#### Identification of candidate absorbed compounds

Based on the grading standards of the Metabolomics Standards Initiative (MSI) [9], the data were compared with a local database and classified. Identified substances were divided into four levels: Level 1: substances in the sample match both MS1, MS2, and retention time (RT) of reference standards in Biotree TCM database; Level 2: substances in the sample match MS1 and MS2 data from public spectral databases; Level 3: Metabolites in the sample match MS1, MS2, and predicted RT from theoretical databases; Level 4: Unknown compounds. Substances at level 1 were screened out as the primary candidate absorbed compounds for subsequent analysis. Compared with the blank rat’s serum, substances present in water extract of HT decoction and HT-containing serum are considered as candidate absorbed compounds.

#### Identification of HT-related genes

SMILES format of candidate absorbed compounds were obtained via PubChem (https://pubchem.ncbi.nlm.nih.gov/). We predicted HT-related genes using SwissTargetPrediction (http://www.swisstargetprediction.ch/) to identify potential targets.

#### Identification of HT-RA targets

We retrieved RA targets by searching the Gene Expression Omnibus (GEO) database (https:/www.ncbinlm.nih.gov/geo/). The RA data microarray GSE55235 was screened, and differentially expressed genes (DEGs) between RA patients and healthy individuals were obtained to identify targets related to RA. We used Venny (v2.1; http://bioinfogp.cnb.csic.es/tools/venny/) to find where the key targets of HT’s main active compounds intersected with RA targets, thereby obtaining the key targets of these active compounds in their treatment of RA.

#### PPI network analysis and drug-target-disease network analysis

We imported the intersecting targets of HT and RA into STRING database (https://string-db.org) to construct a protein–protein interaction (PPI) network. PPI network analysis of candidate genes was performed using the STRING database with the following parameters: Homo sapiens as the species, minimum interaction confidence score > 0.7, and isolated nodes hidden. The resulting PPI network was subsequently subjected to topological analysis via the CytoNCA plugin within the Cytoscape software (version 3.10.1). Six centrality metrics were employed for node prioritization: betweenness centrality (BC), closeness centrality (CC), degree centrality (DC), eigenvector centrality (EC), local average connectivity (LAC), and network centrality (NC). Key targets were identified by selecting nodes that simultaneously exceeded the median values of all six metrics. This multi-metric screening strategy aimed to capture nodes with both global and local topological significance within the network.

#### Pathway enrichment analysis

Using DAVID database (https://david.ncifcrf.gov/), we conducted Gene Ontology (GO) and Kyoto Encyclopedia of Genes and Genomes (KEGG) pathway analyses. Target genes selected for the treatment of RA with HT were input along with official gene symbols and a list of chosen genes. The species was specified as *Homo sapiens*. We performed functional and pathway enrichment analyses (FEA and PEA, respectively) on targets. For KEGG enrichment analysis, we set a threshold of *P* < 0.05 and *Q* < 0.05 for screening.

#### Molecular docking

We scored the binding affinity of HT’s components to target proteins using PyMOL (Schrödinger, Mannheim, Germany) and visualized these scores in Autodock Vina v4.2 (https://vina.scripps.edu/). The protein structure was downloaded from the UniProt database (uniprot.org/), and small-molecule structures were downloaded from PubChem and saved as Protein Data Bank format. Each site on the macromolecular structure was represented using “pseudo-atoms” for docking calculations, and the active site with the highest number of amino acids was selected for molecular docking. Next, we prepared the selected target protein, including removing duplicate units, adding hydrogen and charges, selecting a force field, and deleting unnecessary water molecules. Then, the 20 most significant active ingredient ligands were subjected to energy minimization to determine the docking site of the protein. For evaluation, we selected the scoring function “alpha spheres and excluded volumes” (ASE), which is based on ligand–receptor binding affinity, thereby obtaining the docking score of each compound with the receptor and negative docking score indicates binding.

### In vitro experiment

#### Cell viability assay

We cultured RAW264.7 cells in α-minimum essential medium (α-MEM) with 10% fetal bovine serum (FBS) at 37 °C and 5% CO_2_. A cell viability assay was performed using the CCK-8 Kit. In brief, we seeded RAW264.7 cells in a 96-well plate at a density of 20,000 cells/mL for 24 h. Following treatment with various concentrations of HT-containing serum (0%, 2.5%, 5%, 10%, and with blank rat serum added for a total serum concentration of 10%) for 72 h at 37 °C, cells were added to wells with 10 μL CCK-8 solution and 90 μL α-MEM per well and incubated for 1 h. Subsequently, we measured optical density (OD) at 450 nm using Eon microplate spectrophotometer (BioTek Instruments, Inc., Santa Clara, USA). Cell viability was calculated as follows:

$$\left( {{{{\text{OD }} - {\text{ OD}}\,\left( {\text{blank well}} \right)} \mathord{\left/ {\vphantom {{{\text{OD }} - {\text{ OD}}\,\left( {\text{blank well}} \right)} {{\text{OD}}\,\left( {{\text{control}}} \right)\, - \,{\text{OD}}\,\left( {\text{blank well}} \right)}}} \right. \kern-0pt} {{\text{OD}}\,\left( {{\text{control}}} \right)\, - \,{\text{OD}}\,\left( {\text{blank well}} \right)}}} \right)\, \times \,{1}00\%$$

#### Osteoclastic differentiation

To generate Osteoclasts (OCs), RAW264.7 were cultured in α-MEM with 10% total serum presence of 50 ng/mL of RANKL and 30 ng/mL of M-CSF at a density of 10,000 cells/mL for 5 days. For control group, RAW264.7 were cultured with 30 ng/mL of M-CSF without RANKL; For positive control, Zol were added and worked at 5μmol/mL.

#### Bulk RNA sequencing

On day5 of osteoclastic differentiation, we extracted total RNA using TRIzol according to the manufacturer's instructions. Subsequently, we conducted bulk transcriptome RNA sequencing (RNA-seq) using the Illumina Novaseq^™^ 6000 sequencer (Lianchuan Biotechnology Co., Ltd., Hangzhou, China) with 2 × 150 bp paired-end sequencing (PE150), operational procedures and bioinformatics analysis were following vendor’s recommendations.

#### Metabolomics for osteoclasts

For untargeted metabolomics analyses, LC/MS and untargeted metabolomics raw data analyses were performed at Lianchuan Biotechnology Co., Ltd. (Hangzhou, China) as previously described [10]. Briefly, chromatographic separations were performed using an UltiMate 3000 UPLC System (ThermoFisher Scientific, Bremen, Germany), and TripleTOF 6600 quadrupole time-of-flight (Q-TOF) system (SCIEX, Framingham, USA) was used to detect metabolites eluted from the column, operating the system in both positive and negative ion modes. Bioinformatic analysis was performed using the OmicStudio (https://www.omicstudio.cn/tool).

### Western blotting

Western blotting were performed as previously described [4].

### Data analysis

Data are presented as mean ± standard error of the mean (SEM). We determined statistical differences using Student’s *t* test. For multiple-group comparisons, one-way analysis of variance (ANOVA) was applied, followed by the least significant difference (LSD) when equal variance was assumed or Dunnett’s T3 when equal variance was not assumed. All statistical analyses were conducted using GraphPad Prism 9.6 (GraphPad Software, San Diego, USA) and SPSS version 26.0 (IBM Corp., Armonk, NY, USA).

## Results

### HT reduced inflammatory arthritis in AIA rats

**Fig. 1:**
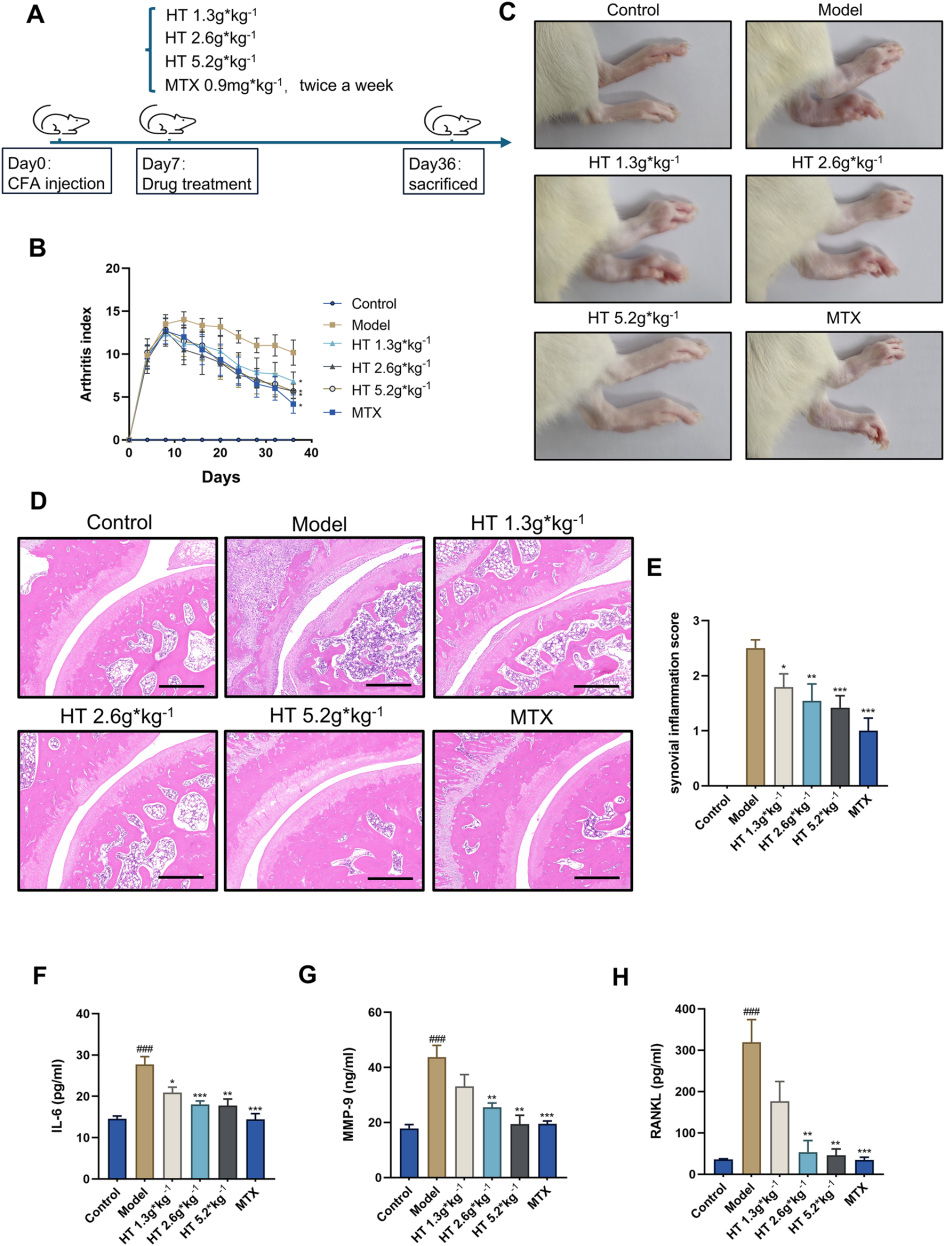
HT reduced inflammatory arthritis in AIA rats. **A** Schematic of animal administration regimen. **B** Total AI score in each group (n = 6). **C** Representative images of swollen joints in each group. **D**, **E** H&E staining and synovial inflammation scores of joints in each group. **F**–**H** Serum levels of IL-6, MMP-9, and RANKL in each group. Data are shown as mean ± SEM. Scale bar = 500 μm. ###*P* < 0.001 vs. control group; *P < 0.05, **P < 0.01, and ***P < 0.001 vs. model group

Rats reached the peak of inflammation on day 8 after initial immunization, were treated with different doses of HT or MTX, and were sacrificed on day 36. Figure 1A illustrates the specific procedure. The BW of the AIA rats decreased compared with rats in control group after initial immunization (Fig. S1A). After drug administration, the BW growth rate gradually returned to normal in each of the HT dosage groups and in the MTX group. In addition, CFA-induced liver, spleen, and kidney indices were significantly lower in the treatment groups than in the model group, suggesting that inflammation-induced pressure on rats’ immune systems and metabolic organs was reduced after treatment (Fig. S1B–G). The degree of swelling in the affected areas and Arthritis Index (AI) scores reflected the severity of inflammation. Compared with the model group, joint swelling was significantly reduced in rats in the 1.3 g × kg^−1^, 2.6 g × kg^−1^, and 5.2 g × kg^−1^ HT groups, as well in those in the MTX group (Fig. 1B). Ankle joint swelling in these rats was also reduced (Fig. 1C). In the model group, synovial tissue of ankle joints exhibited multilayered synovial membranes with inflammatory infiltration, disordered arrangement of joint structures, and damage to the joint surface. Compared with the model group, synovial tissue hyperplasia and inflammatory infiltration were alleviated in the 1.3 g × kg^−1^, 2.6 g × kg^−1^, and 5.2 g × kg^−1^ HT groups and the MTX group (Fig. 1D, E).

Obvious cartilage damage and bone erosion occur during the persistent progression of RA, which can eventually result in joint deformity and disability [11]. After HT treatment, levels of serum inflammatory and osteoclastic-differentiation–related cytokines in AIA rats were also improved. Compared with the control group, levels of the serum inflammatory cytokine IL-6 and OC-related factors MMP-9 and RANKL were significantly elevated in the model group. After HT administration, levels of serum IL-6, MMP-9, and RANKL decreased in the 1.3 g × kg^−1^, 2.6 g × kg^−1^, and 5.2 g × kg^−1^ HT groups and the MTX group compared with the model group (Fig. 1F–I). Taken together, these results indicate that HT significantly reduced joint inflammation in AIA rats and has clinical potential to reduce bone erosion in arthritis.

### HT treatment reduced subchondral bone deterioration and articular cartilage degeneration in AIA rats

**Fig. 2:**
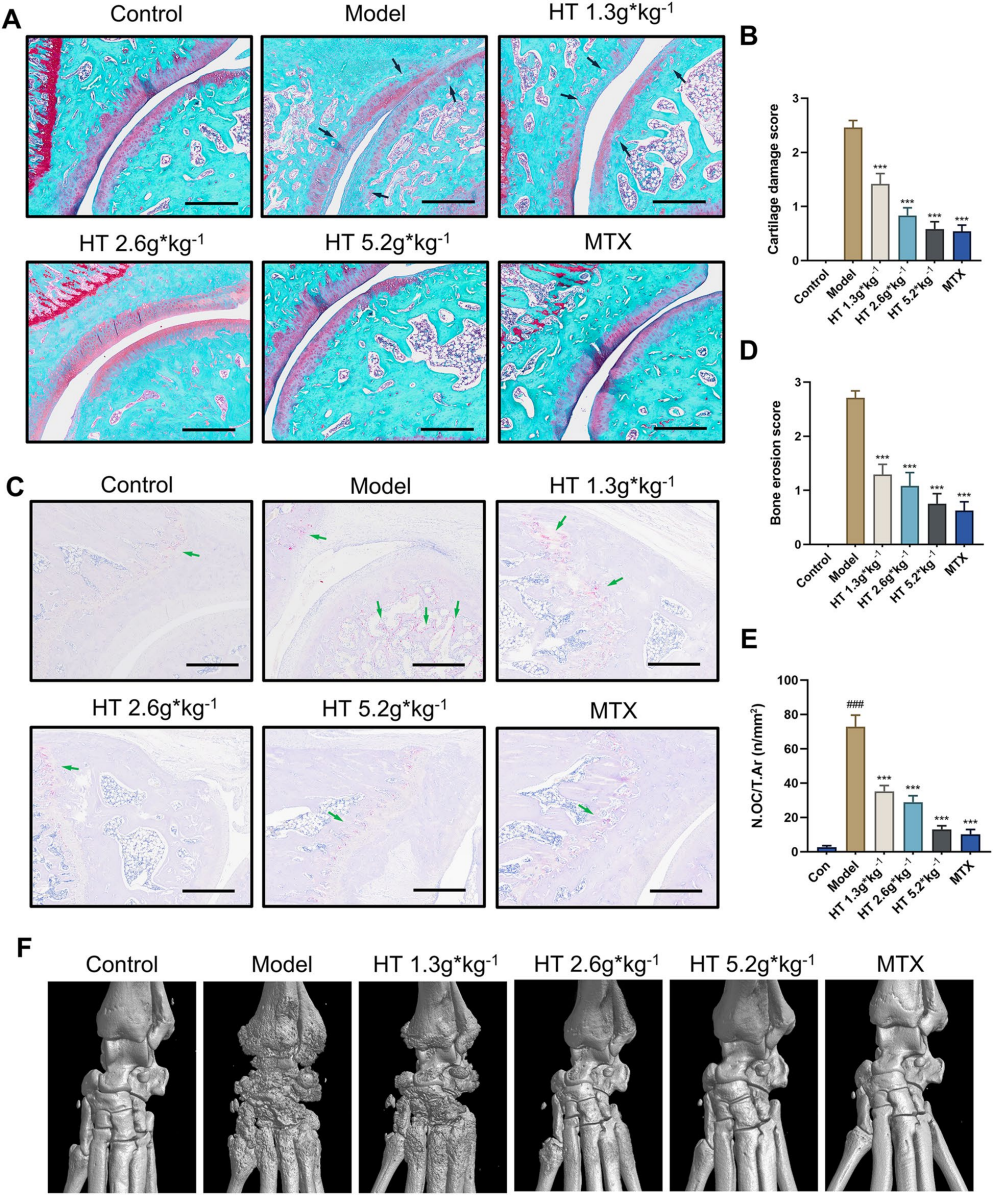
HT treatment reduced subchondral bone deterioration and articular cartilage degeneration in AIA rats. **A**, **B** Safranin O/Fast Green staining of ankle joint tissue sections in AIA rats (n = 6) and cartilage damage score. Black arrowheads indicate proteoglycan loss area. **C**–**E** TRAP staining of ankle joint tissue sections in AIA rats (n = 6) and bone erosion score. Number of OCs per tissue surface (N.OC/T.Ar). Green arrowheads indicate eroded bone tissue with OCs. **F** Representative micro-CT images of ankle joints. Data are shown as mean ± SEM. Scale bar = 500 μm. ###*P* < 0.001 vs. control group; *P < 0.05, **P < 0.01, and ***P < 0.001 vs. model group

To further elucidate the protective effects of HT against cartilage damage and bone erosion in AIA rats, we performed Safranin O/Fast Green and TRAP staining as well as micro-CT analysis of hind paw tissues. The results demonstrated that compared with the model group, HT inhibited cartilage erosion in the subchondral bone of the ankle joint surface and reduced proteoglycan loss (Fig. 2A, B). TRAP staining showed that HT alleviated synovial pannus invasion and partial loss of bone structure and decreased the number of TRAP^+^ OCs in eroded bone tissue (Fig. 2C–E). Micro-CT revealed that AIA model rats displayed rough bone surfaces, loss of bone structure, and erosion of bone tissue. Compared with the model group, bone destruction decreased in a dose-dependent manner in the HT-treated groups (Fig. 2F). Therefore, HT treatment efficiently reduced subchondral bone deterioration and articular cartilage degeneration in inflamed joints.

### Identification of HT’s major absorbed components

**Fig. 3:**
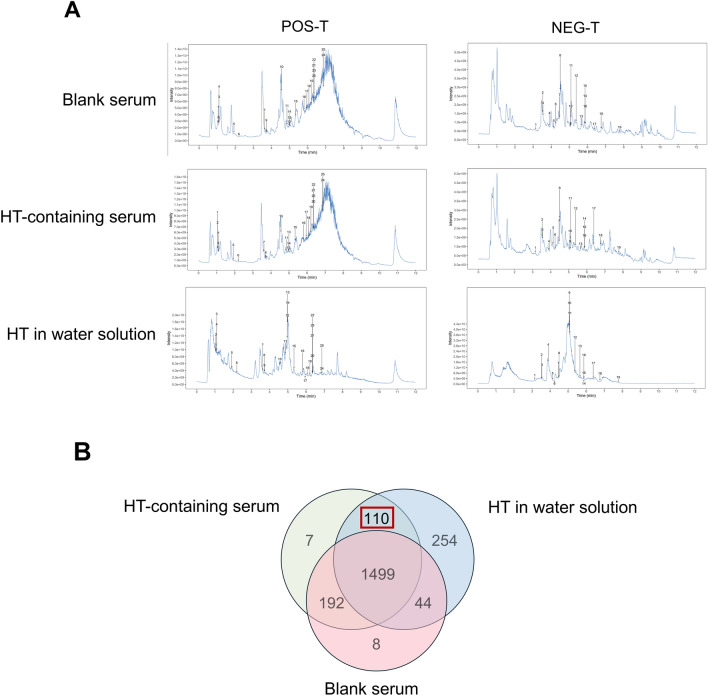
Identification of the major absorbed components of HT. **A** Mass spectrum of HT in water solution, HT drug-containing serum, and blank serum. **B** Venn diagram showing different numbers of components

To elucidate the specific mechanisms underlying the alleviation of RA inflammation and bone destruction by HT, we conducted a component analysis of HT through mass spectrometry and compared the results of HT drug-containing serum with blank serum. Ultimately, 110 absorbed components of HT were preliminary identified (Fig. 3A, B) and peak number, pubchem IDs, mass spectrometry parameters of the 44 identified level1 components (which in the sample match the MS1, MS2, and RT of the reference standards) from 110 absorbed components of HT are listed in Table.S1, and additional information are included in Supplementary Material. Based on their SMILES formulas, 753 target points of HT action were obtained from the Swiss Target database.

### Network pharmacology investigation of major targets of HT in treating RA

**Fig. 4:**
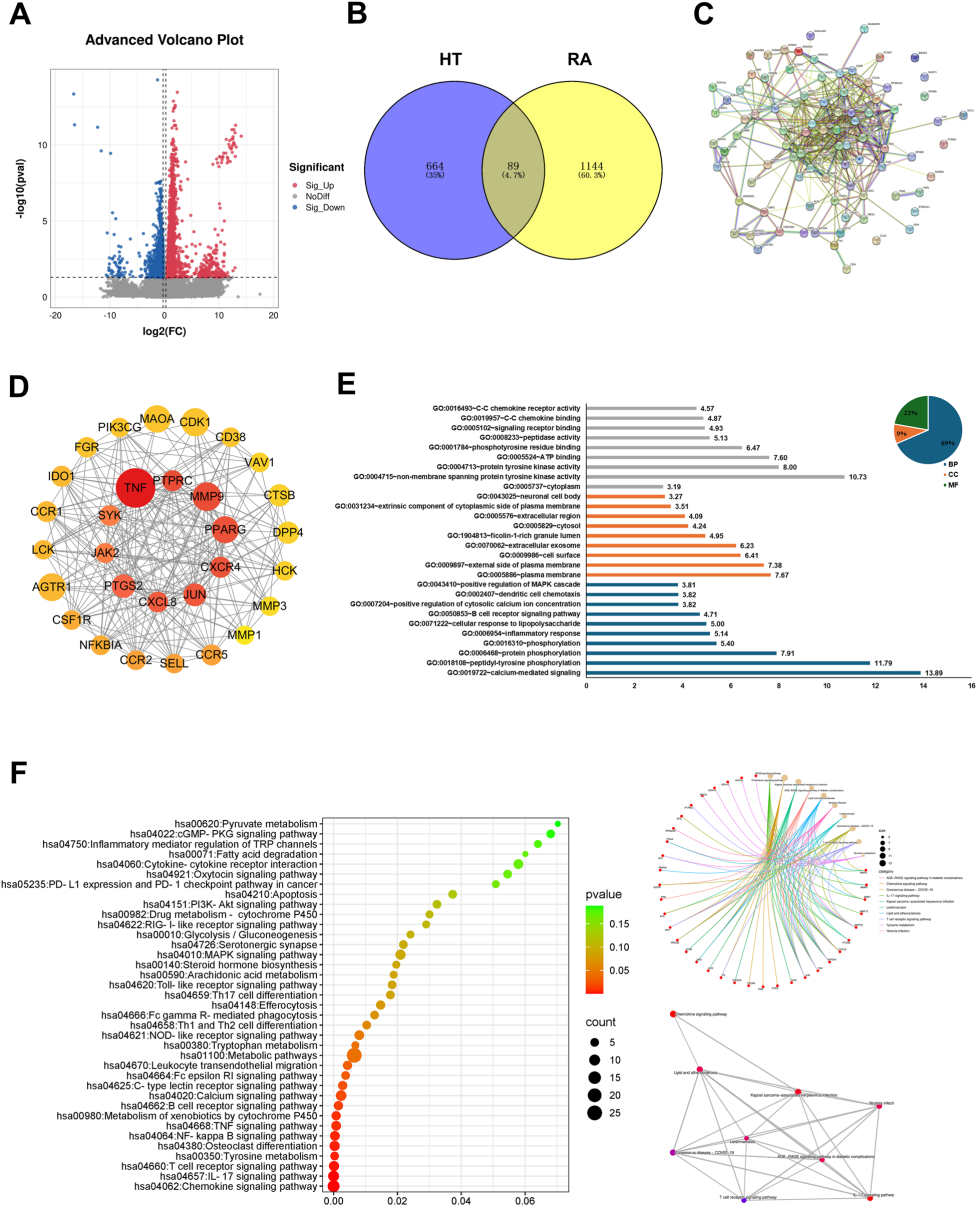
Network pharmacology investigation of major targets of HT in treating RA. **A** RA target intersection volcano map. **B** Venn diagram with RA and HT target genes related to RA from the GEO database. **C** PPI network. **D** Screening for major targets. **E** GO enrichment analysis of RA-HT. **F** KEGG signaling pathway analysis

Based on the intersections of RA DEGs and the main active ingredient targets of HT, PPI results obtained from the STRING database revealed which target proteins played crucial roles in the PPI. We obtained 89 core targets of HT that might act on RA (Fig. 4A–C). Target points with interaction relationships were sorted by degree value. Tumor necrosis factor (TNF), peroxisome proliferator–activated receptor γ (PPARG), prostaglandin-endoperoxide synthase 2 (PTGS2), and MMP-9 had the highest degree values (Fig. 4D). For GO enrichment analysis, multiple Biological Process (BP), Cellular Component (CC), and Molecular Function (MF) terms were screened out using the DAVID. The results indicated that HT’s mechanism of action in treating RA might be associated with various BPs (Fig. 4E). We created a graph of the top 20 pathways ranked by *P*-value (Fig. 4F), with targets significantly enriched in multiple pathways such as IL-17, NF-κB, and B-cell receptor.

### Molecular docking of key components and targets of HT in treating RA

**Fig. 5:**
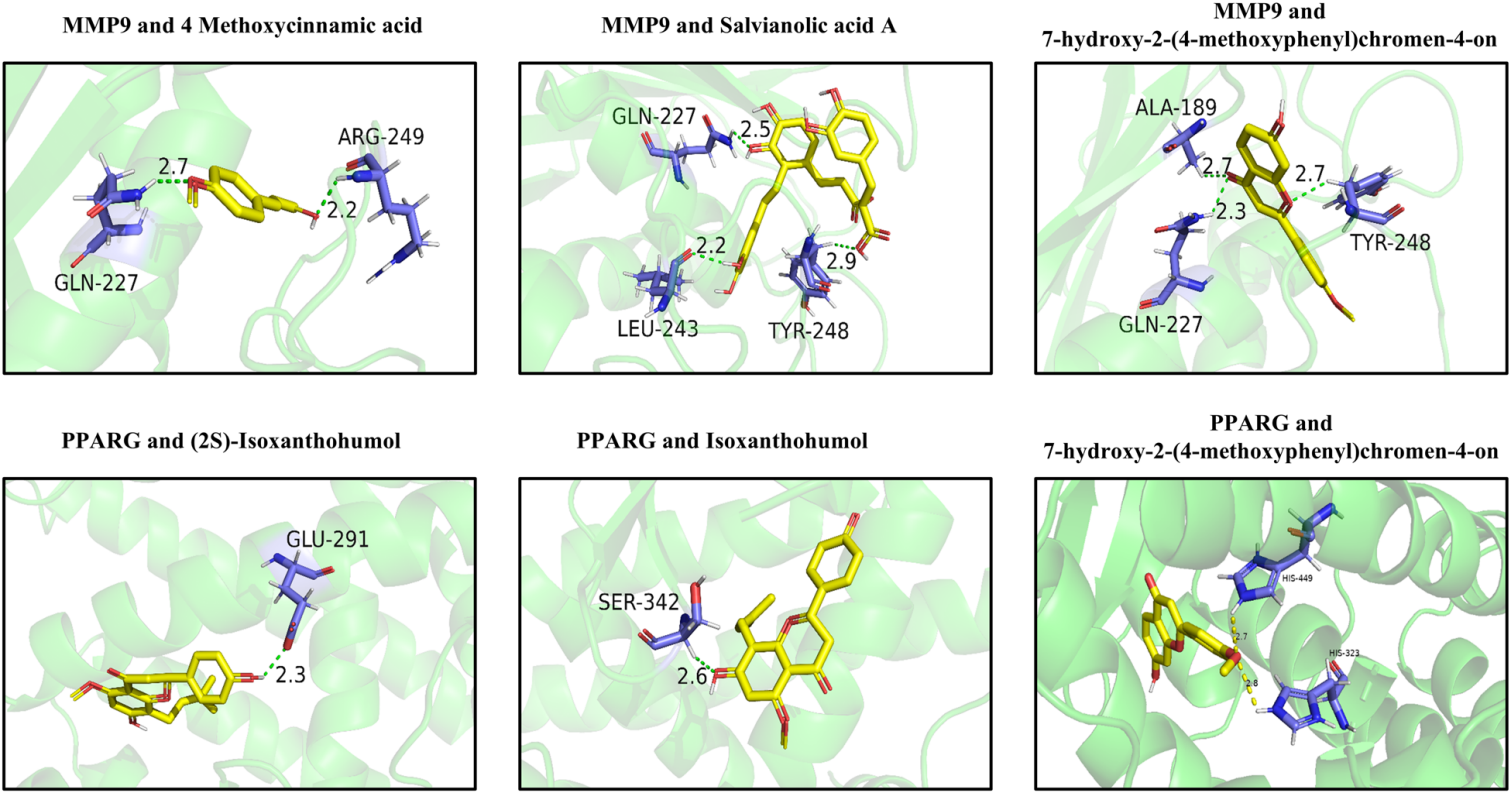
Three-dimensional diagrams demonstrate the molecular docking interactions between key components of HT and therapeutic targets in R

To further validate network pharmacology results of HT-related targets in treating RA, we performed molecular docking validation of major active compounds and their corresponding targets (Table 2). It is generally acknowledged that a binding energy lower than − 5 kcal/mol between a receptor protein and a ligand compound indicates the presence of a confirmed binding affinity [12]. Among them, the typical active components Salvianolic acid A, 4-Methoxycinnamic acid, and 7-hydroxy-2-(4-methoxyphenyl) chromen-4-one (also known as Pratol) exhibited good binding capacity with the typical target MMP9. Moreover, the docking results of Isoxanthohumol, Pratol, and (2S)-Isoxanthohumol with PPARG indicated their ability to bind well with PPARG (Fig. 5). These results suggest that the major active compounds possess significant binding potential towards target gene-related proteins.

**Table 2:** Molecular docking of key components and targets

Targets	Relative to RA	Compounds	Score (kcal/mol)
MMP9	Bone matrix degradation	Salvianolic acid A	−9.2
		4-Methoxycinnamic acid	−7.5
		7-hydroxy-2-(4-methoxyphenyl)chromen-4-one	−10.1
PPARG	Bone metabolism; inflammation	Isoxanthohumol	−7.8
		(2S)-Isoxanthohumol	−7.5
		7-hydroxy-2-(4-methoxyphenyl)chromen-4-one	−7.8
TNF	Inflammation	Isoxanthohumol	−5.8
		(2S)-Isoxanthohumol	−6.6
PTPRC	Immune biomarker	4-Methoxycinnamic acid	−6.8
JUN	JAK/STAT signalling pathway; Inflammation	Griffonilide	−5.3

### HT-containing serum suppressed RANKL-induced osteoclastic differentiation in vitro

**Fig. 6:**
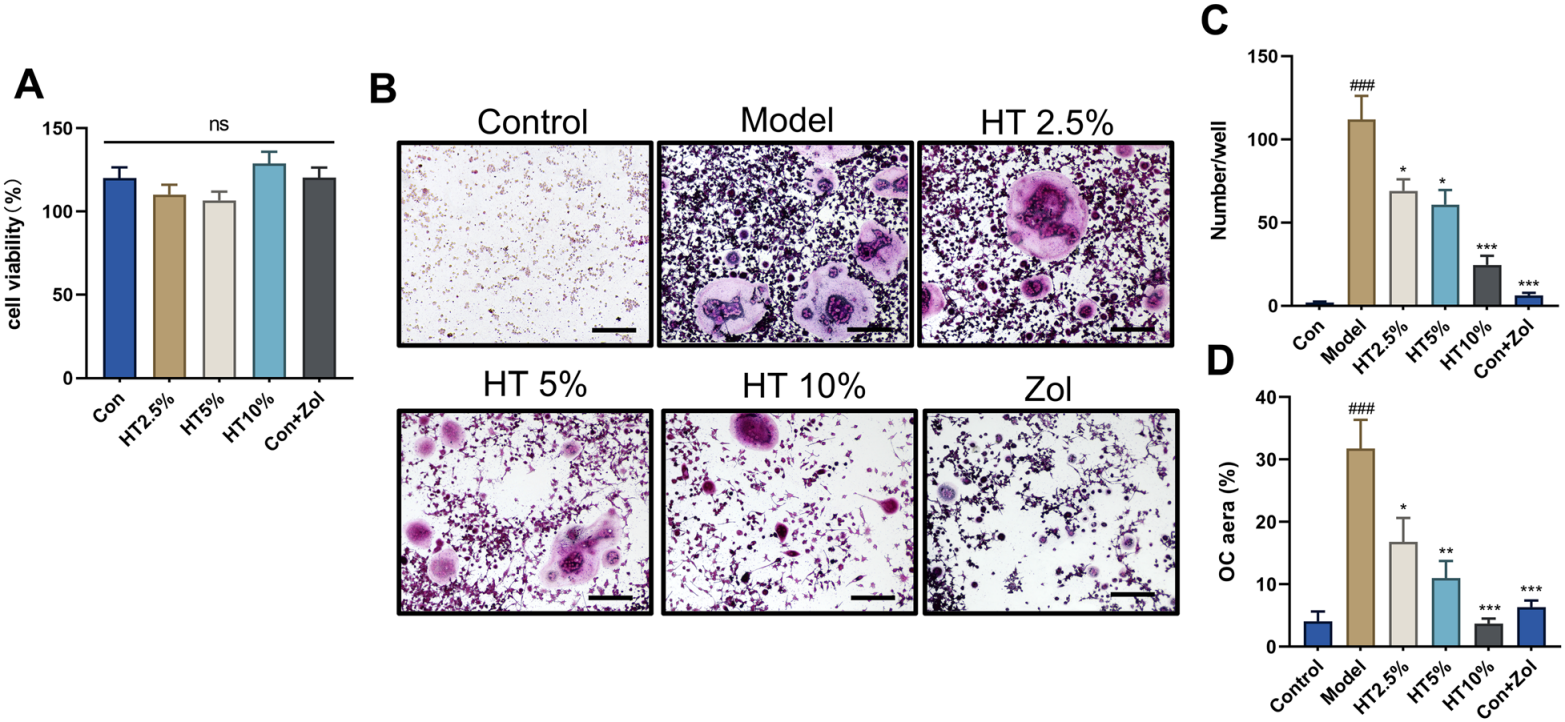
HT-containing serum suppressed RANKL-induced osteoclastic differentiation in *vitro*. **A** CCK-8 essay of HT-containing rat serum and blank serum in which RAW264.7 cells were cultured for 72 h. **B** Representative images. **C**, **D** Quantification of TRAP-stained polynucleated OCs (≥ 3 nuclei) from OC cell culture (n = 6). Data are shown as mean ± SEM. Scale bar = 200 μm. ###*P* < 0.001 vs. control group; *P < 0.05, **P < 0.01, and ***P < 0.001 vs. model group

To further elucidate the effects of HT in RA-related bone erosion, we established a RANKL-induced osteoclastic-differentiation model in vitro. To observe whether the absorbed components of HT could inhibit osteoclastic differentiation in vitro, we prepared blank rat serum and HT-containing serum. CCK-8 assays indicated that HT-containing serum at various concentration gradients had no significant effect on cell viability within 72 h (Fig. 6A). Following osteoclastic induction, multiple cells fused to produce multinucleated giant OCs that showed dark-red TRAP staining, indicating successful induction. With increasing proportions of drug-containing serum, the level of osteoclastic differentiation decreased, and the number of OCs significantly decreased (Fig. 6B–D). Intervention with the positive-control drug Zol acid also demonstrated significant osteoclastic inhibition, indicating that HT-containing serum could inhibit osteoclastic differentiation in vitro*.*

### Validation of the mechanism underlying the effects of HT on osteoclastic differentiation using RNA sequencing

**Fig. 7:**
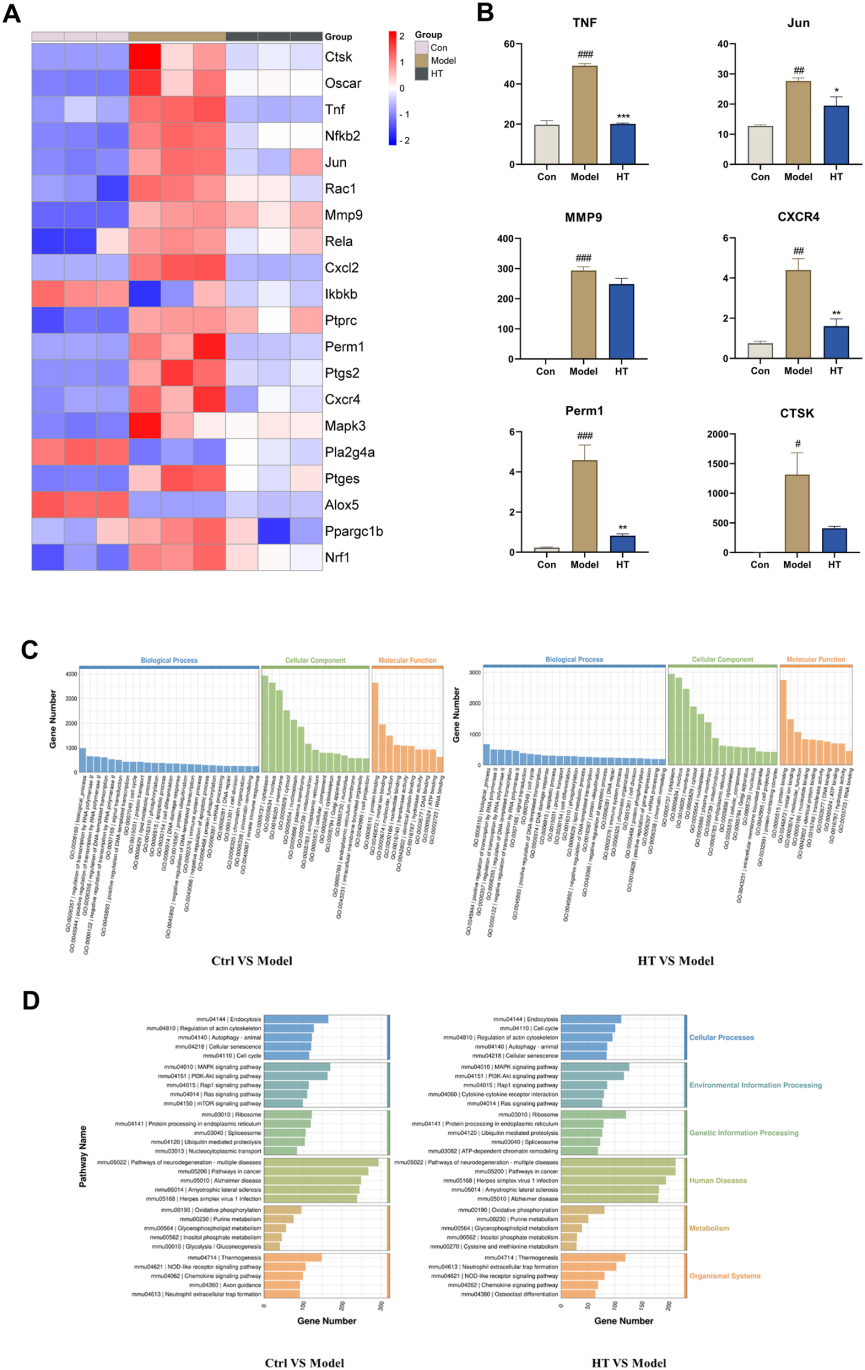
RNA-seq was used to validate the mechanism underlying the effects of HT on osteoclastic differentiation. **A** Heatmap of gene expression in HT-treated osteoclasts. **B** Expression of *Tnf*, *Mmp-9*, *Jun*, *Cxcr4*, *Pparg*, and *Ctsk* in RNA-seq. **C**, **D** GO and KEGG enrichment analyses. Data are shown as mean ± SEM. ###*P* < 0.001 *vs.* control group; *P < 0.05, **P < 0.01, and ***P < 0.001 vs. model group

RNA-seq on the Illumina sequencing platform enables researchers to study the entire RNA of cells in a specific functional state with high precision and accurate quantitative and qualitative detection of gene expression. To validate our network pharmacology results and identify the mechanisms underlying the effects of HT on RA-related bone erosion, we used RNA-seq to explore the role of differential gene expression in HT-containing serum’s regulation of OCs. The results showed that HT-containing serum reduced expression levels of genes related to osteoclastic differentiation, and target genes in the HT–RA PPI network were validated. HT treatment decreased the expression of such targets as *Tnf*, *Mmp-9*, protein tyrosine phosphatase receptor type C (*Ptprc*), *Jun*, and chemokine (C-X3-C motif) receptor 4 (*Cxcr4*; Fig. 7A, B). GO and KEGG analysis showed that the main pathways included the MAPK signaling pathway, PI3K/Akt signaling pathway, and osteoclastic differentiation (Fig. 7C, D). In conclusion, transcriptome expression of target genes was generally consistent with network pharmacology results.

### Metabolomics validate mechanism of HT in osteoclast differentiation

**Fig. 8:**
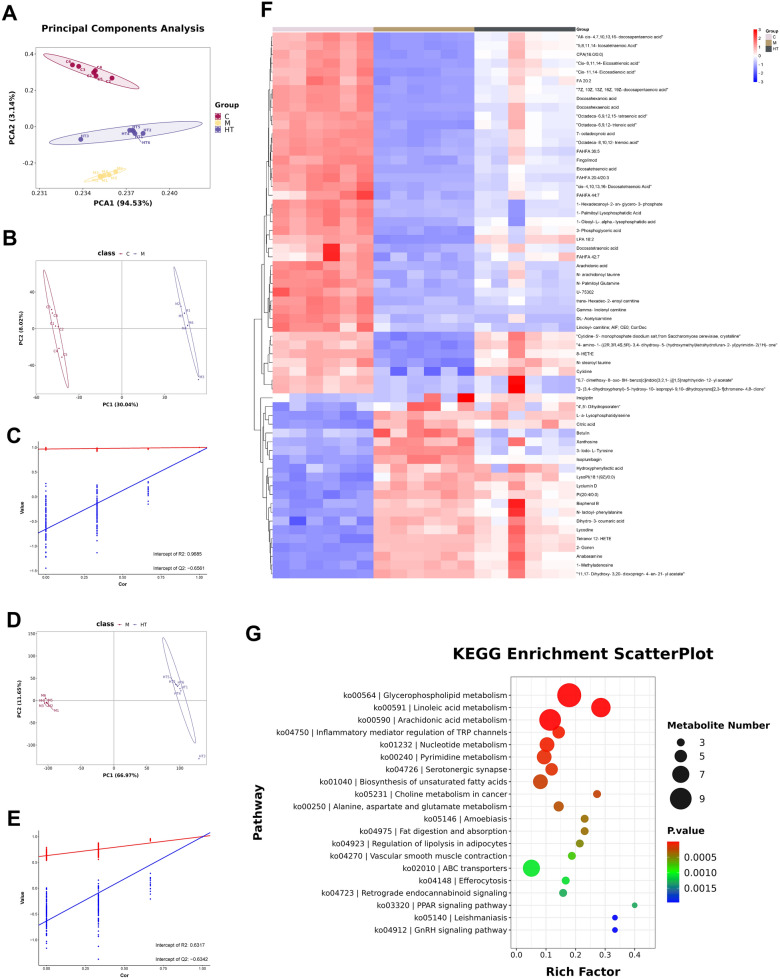
Metabolomics was used to validate the mechanism of HT in osteoclastic differentiation. **A** PCA score plot among the control, model, and HT groups. **B**, **C** OPLS-DA score plot between the control and model groups and the corresponding coefficients of loading plots. **D**, **E** OPLS-DA score plot between the model and HT groups and the corresponding coefficients of loading plots. **F** Heatmap of differential metabolites in HT-treated OCs. **G** KEGG analysis

Metabolic reprogramming in osteoclastic differentiation plays an important role in bone degradation in RA [13, 14]. To further investigate the effects of HT-containing serum on OCs, we used untargeted metabolomics to compare changes in metabolites, analyzing the metabolic effect of HT-containing serum on the osteoclastic-differentiation process. PCA and OPLS-DA of metabolite profiles showed significant differences among the control, model, and HT groups. Moreover, after HT-containing serum intervention, osteoclastic differentiation process was suppressed. the HT group shifted toward the control group (Fig. 8A–E). By comparing the control and model groups, as well as the model and HT groups, we identified a total of 62 different metabolites with VIP values > 1 and *P*-values < 0.05 (FC > 1.2 or < 0.8; Table S2). Compared with the model group, levels of elevated metabolites such as citric acid were reduced in the HT group, while those of arachidonic acid (AA), fingolimod, and other metabolites were increased (Fig. 8F). In addition, KEGG and GO pathway analyses enriched multiple pathways, including AA metabolism, glycerophospholipid metabolism, pyruvate metabolism, and the peroxisome proliferator–activated receptor (PPAR) pathway (Fig. 8G).

### Integrated multiomics and network pharmacology analysis

**Fig. 9:**
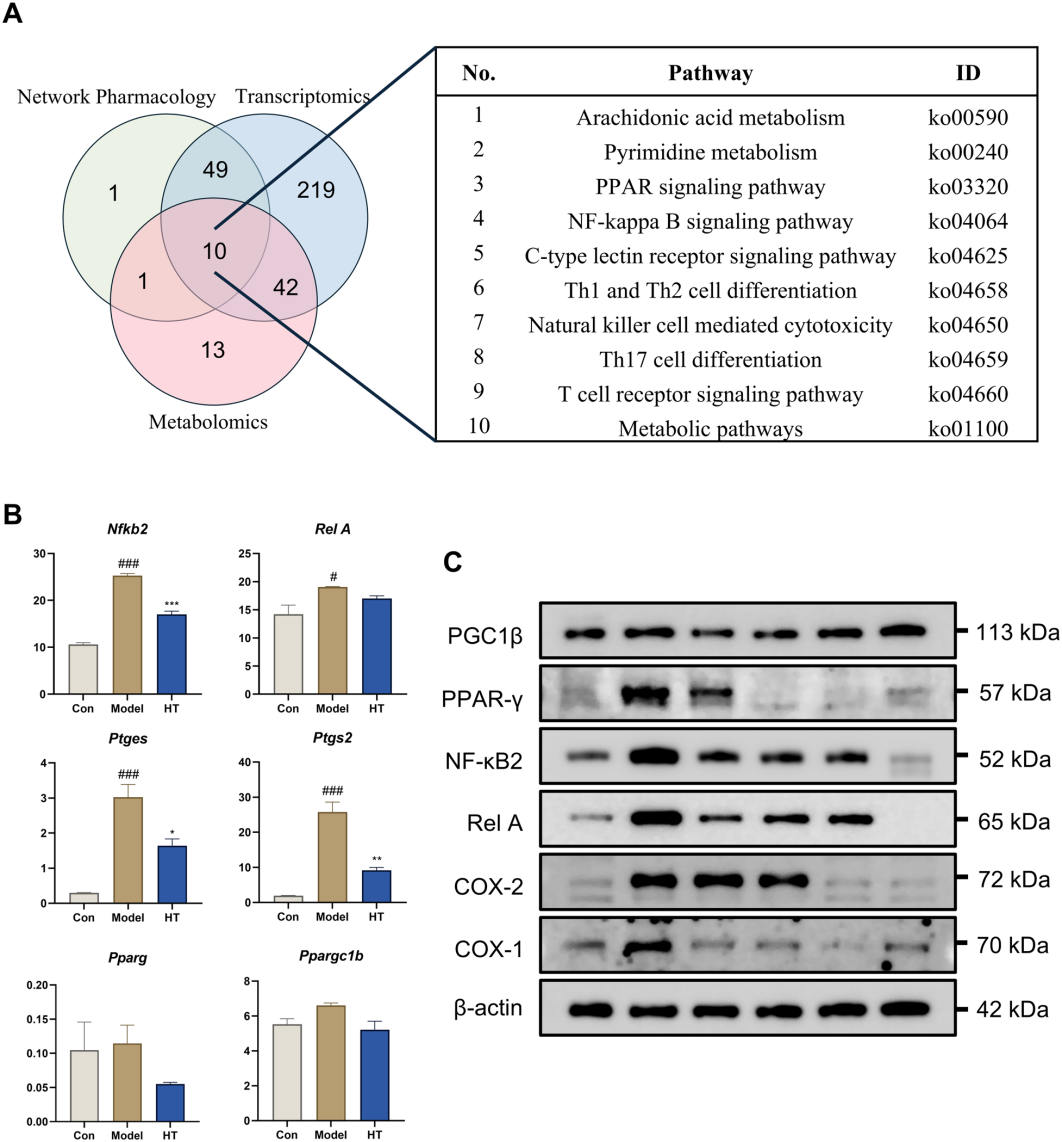
Integrating metabolomics, transcriptomics and network pharmacology analysis. **A** Venn diagram of metabolomics, transcriptomics and network pharmacology and top 10 pathways. **B** Expression of key factor level of PPAR pathway, AA metabolism, and NF-κB pathway in RNA-seq. **C** Western blotting band of key factor level PPAR pathway, AA metabolism, and NF-κB pathway. Data are shown as mean ± SEM. #*P* < 0.05, ###*P* < 0.001 vs. control group; *P < 0.05, **P < 0.01, and ***P < 0.001 vs. model group

By integrating metabolomics, transcriptomics, and network pharmacology, we were able to elucidate the mechanism by which HT reduced bone erosion in RA from a multi-component, multi-target, and multi-effect perspective. Figure 9A illustrates the intersections and related pathways influenced by the results of these combined analyses. BPs such as AA metabolism, NF-κB pathway, PPAR pathway were enriched, and the key factor of AA metabolism, NF-κB pathway, PPAR pathway show that HT-containing serum intervention affected these pathways in OCs (Fig. 9B). WB confirm that affection at protein level (Fig. 9C). These results suggest that HT could ameliorate OC-induced bone erosion accompanying RA through multiple biological processes.

## Discussion

Bone erosion is a hallmark of RA, caused by synovial hyperplasia and inflammation-related cells enhancing osteoclastic differentiation and thus resulting in joint dysfunction and disability. Aging, sexual factors, genetic factors, and long-term glucocorticoid treatment are also risk factors for bone degradation in RA [2, 15]. Current therapies, like Disease-Modifying Antirheumatic Drugs (DMARDs), inhibit the progression of bone destruction in the majority of patients with RA, but in certain cases the response to even multiple DMARDs is inadequate, and it is thus difficult to completely prevent bone destruction. Therefore, we urgently need to find new therapy, and elucidate the cellular and molecular network underlying bone erosion in RA.

TCM decoctions have been widely used in China for centuries and have demonstrated clinical efficacy in various diseases, including RA. Of these, the herbal decoction HT has been used as a complementary treatment for RA, with prior studies highlighting its anti-RA properties. HT is a formulation developed by Professor Qingchun Huang under the guidance of the TCM theory that "blood stasis is the key of rheumatoid arthritis." It integrates the clinical experiences of Academician Liu Liang and Professor Zhu Liangchun, combining an optimized selection from the traditional Chinese formulas: Wutou Decoction and Shaoyao Gancao Decoction, with blood-activating and collateral-dredging herbs Chuan Shan Long (*Dioscorea nipponica*) and Danshen (*Salvia miltiorrhiza*). In this study, we specifically investigated the effects of HT on bone erosion in experimental arthritis models. Our findings revealed that HT significantly reduced serum levels of bone erosion–related factors, mitigated articular cartilage damage, and attenuated bone erosion, suggesting its potential to ameliorate RA-related bone destruction.

Due to their complex composition, studies of Chinese herbal decoctions remain challenging. To address this, we used serum pharmacochemistry to identify and screen major absorbed components of HT, such as Salvianolic acid A, Isoxanthohumol, 4-Methoxycinnamic acid, and Pratol, and constructed a component-target-pathway network based on the major absorbed components using network pharmacology analysis, and several key targets of RA have also been confirmed by molecular docking that exhibiting strong binding affinity with components of HT, like TNF and MMP-9, the inflammation and bone destruction biomarkers. NF-κB and JNK pathways also play the important role in RA, and previous studies have shown that Salvianolic acid A and Isoxanthohumol can improve joint inflammation through NF-κB [16–18], and Pratol affects the NF-κB and JNK pathways [19], Those findings suggest that HT may alleviate RA through these targets and their associated pathways.

Recent research has emphasized the critical role of enhanced osteoclastic differentiation and function, driven by metabolic alterations, in RA-related bone erosion [20, 21], Therefore, inhibiting osteoclastic differentiation and function has emerged as a promising therapeutic strategy for alleviating RA-induced bone damage [22]. Our network pharmacology analysis analysis revealed that HT significantly affected the osteoclastic-differentiation pathway, prompting us to construct an in vitro osteoclastic-differentiation model to evaluate these effects. The results demonstrated that HT effectively inhibited RANKL-induced osteoclastic differentiation, further underscoring the multi-target and multi-effect nature of TCM decoctions.

Multiomics analyses conducted in vitro provide a new research approach, allowing us to focus more on the multi-effects of HT and the potential mechanisms by which it inhibits RA-related bone erosion via inhibition of osteoclastic differentiation. By integrating multiomics results conducted in vitro, we enriched pathways such as the PPAR pathway, AA metabolism, and NF-κB pathway, which were generally consistent with predictions from network pharmacology. The results indicated that HT exhibited the characteristics of “multiple components, multiple targets, and multiple effects” presenting a multifaceted mechanism of action for treating RA. One of these facets, osteoclastic differentiation, was related to joint inflammation and immunity; multiple targets were significantly enriched in the osteoclastic-differentiation pathway.

The NF-κB pathway plays a crucial role in inflammation and osteoclastic differentiation. Its primary effector ligand, RANKL, is a well-known osteoclastogenic factor that is widely used to induce osteoclastic differentiation in vitro. Inhibitors of RANKL, such as Zol, have been clinically applied in the treatment of osteoarthritis to prevent bone loss caused by the long-term use of glucocorticoids due to their significant inhibitory effects on OCs [23]. Our results showed that HT could effectively inhibit RANKL-induced osteoclastic differentiation, suggesting that HT might inhibit RA-related bone destruction through NF-κB pathway.

The PPAR signaling pathway has been identified as a potential inhibitory target for OCs [24, 25]. PPAR-γ is a key regulator of osteoclastogenesis-related factors such as c-Fos and PGC1-β, promoting the differentiation and formation of OCs. In our in vitro studies on OCs, PPAR pathway–related genes and metabolism were inhibited after HT intervention in RANKL-induced OCs. Interestingly, the PPAR signaling pathway inhibits the function of macrophages and suppresses pannus formation via apoptosis of macrophages, synovial cells, and endothelial cells, reducing inflammation in RA [26, 27]. Activation of this pathway is considered a potential therapeutic approach to inhibiting synovial inflammation and pannus neovascularization in RA [28], suggesting that the PPAR signaling pathway exhibits different effects in different cells. The heterogeneity of different cells requires further investigation.

Several enzymes of AA metabolism, such as cyclooxygenases-2 (COX-2) are well-recognized targets for anti-inflammatory drugs that can reduce symptoms of inflammation in rheumatic diseases [29]. AA is primarily metabolized by COX, lipoxygenase, and P450 and then converted into various metabolites that trigger inflammatory responses, exacerbating synovial inflammation in joints, which in turn leads to bone erosion in the joints [30]. AA metabolism can also directly affect the metabolic process of joint OCs [31]; its inhibition can lead to accumulation of AA, which can inhibit osteoclastic differentiation induced in RAW264.7 cells and bone marrow–derived macrophages [32]. In this study, we also observed accumulation of AA. Therefore, AA metabolism is another possible mechanism by which HT inhibits bone destruction in RA.

This study has several limitations. First, metabolic transformations of HT components over time after entering the bloodstream were not fully characterized, and serum pharmacochemical analysis did not fully account for potential synergistic components in the HT, such as unabsorbed prodrugs or bioactive metabolites, future studies should focus on how to characterize the full spectrum of HT-derived compounds in systemic circulation. Second, AIA model does not recapitulate the chronic progression and immune complexity of RA, and the RANKL-induced osteoclastic-differentiation model did not entirely replicate the complex process of RA-related bone erosion too. Additionally, variations in omics analysis software and sequencing platforms might have introduced discrepancies in the results.

In summary, HT reduced joint inflammation and inhibited osteoclastic differentiation through multiple mechanisms, including modulation of the NF-κB pathway, PPAR signaling pathway, and AA metabolism. These findings highlight HT’s potential as a multi-target therapeutic agent for RA-related bone destruction. By integrating network pharmacology with multiomics, we elucidated the mechanisms underlying HT’s anti-RA effects, providing a foundation for further research.

## Conclusion

This study confirmed the beneficial effects of HT in experimental arthritis and explored the specific mechanisms involved. HT inhibited osteoclastic differentiation through multiple targets and pathways to alleviate RA-related bone erosion, providing a potential therapeutic strategy for preventing this issue.

## Supplementary Information

Supplementary material 1.

Supplementary material 2.

## Data Availability

Data presented in the main manuscript or Supplementary material. Additional data which were not included will be provided on reasonable request.

## Abbreviations

RA: Rheumatoid arthritis
HT: Huayu-Tongbi decoction
TCM: Traditional Chinese Medicine
AIA: Adjuvant-induced arthritis
ELISA: Enzyme-linked immunosorbent assay
micro-CT: Micro-computed tomography
RANKL: Receptor activator of nuclear factor κ-β ligand
MMP-9: Matrix metalloproteinase-9
LC–MS/MS: Liquid chromatography with tandem mass spectrometry
GO: Gene ontology
KEGG: Kyoto encyclopedia of genes and genomes
PPI: Protein–Protein interaction
PCA: Principal component analysis
OPLS-DA: Orthogonal partial least squares discriminant analysis
AA: Arachidonic acid
PPAR: Peroxisome proliferator-activated receptor
NF-κB: Nuclear factor κ-light-chain-enhancer of activated B cells
JAK/STAT: Janus-associated kinase/signal transducer and activator of transcription
TRAP: Tartrate-resistant acid phosphatase
H&E: Hematoxylin and eosin
CCK-8: Cell counting kit-8
M-CSF: Macrophage colony-stimulating factor
Zol: Zoledronic acid
GEO: Gene expression omnibus
DEGs: Differentially expressed genes
FEA: Functional enrichment analysis
PEA: Pathway enrichment analysis
SEM: Standard error of the mean
ANOVA: Analysis of variance
LSD: Least significant difference
RNA-seq: RNA sequencing
LC/MS: Liquid chromatography/mass spectrometry
Q-TOF: Quadrupole time-of-flight
VIP: Variable importance in projection
FC: Fold change
BPs: Biological processes
CC: Cellular components
MF: Molecular functions
TNF: Tumor necrosis factor
MTX: Methotrexate
PPARG: Peroxisome proliferator-activated receptor γ
PTGS2: Prostaglandin-endoperoxide synthase 2
PTPRC: Protein tyrosine phosphatase receptor type C
JUN: Jun proto-oncogene
CXCR4: C-X-C motif chemokine receptor 4
CTSK: Cathepsin K
