# Supplementary material for: Multi-omics and network pharmacology reveal Huayu-Tongbi decoction reduced arthritis-related bone erosion
Source: Chin Med. 2025 Jul 2;20:100. doi: 10.1186/s13020-025-01159-1 (PMC12220612; doi:10.1186/s13020-025-01159-1)
Supplement: Supplementary file 1 — Supplementary material 1. [file 13020_2025_1159_MOESM1_ESM.docx]

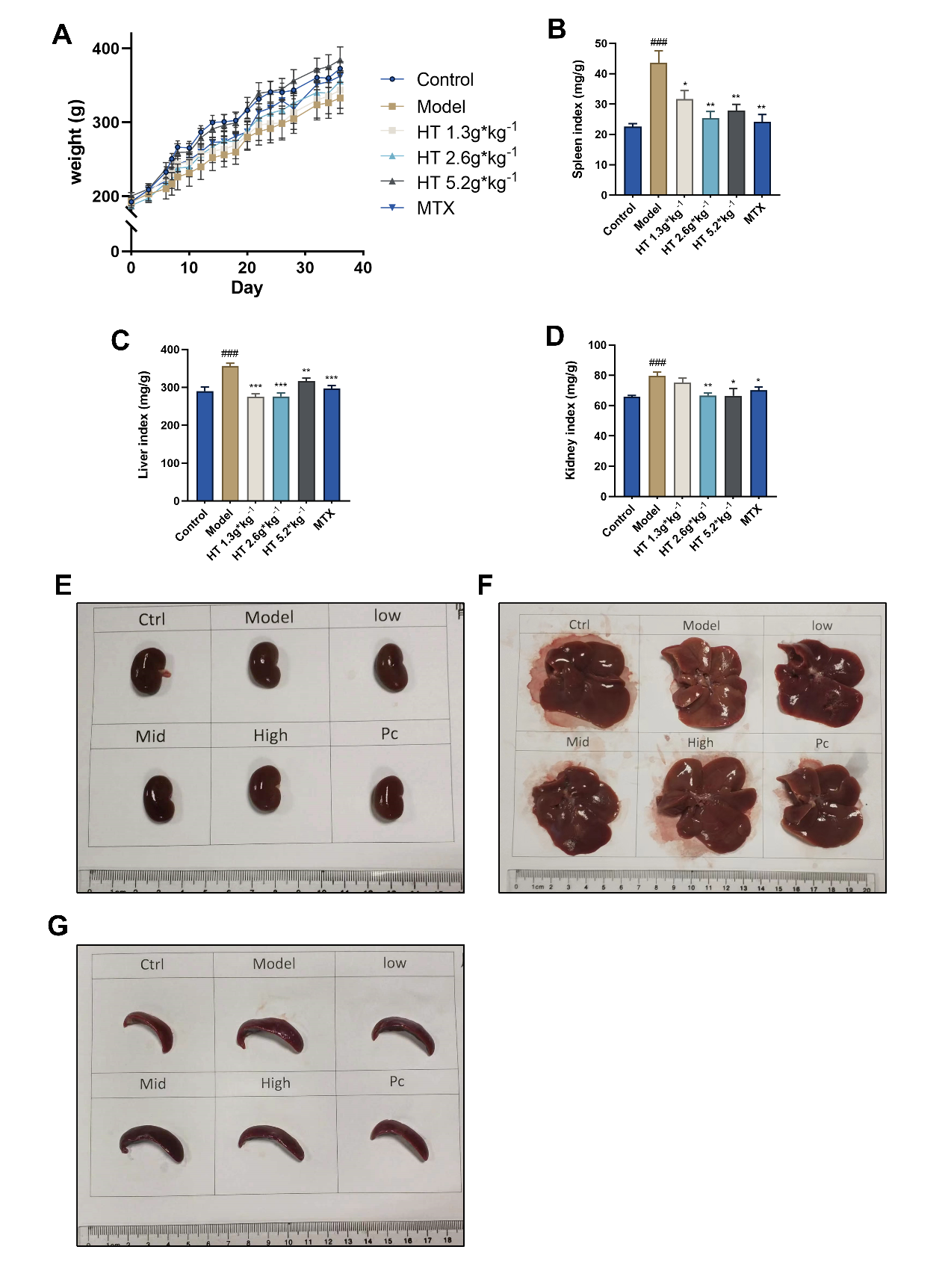
Figure.S1 Effect of HT on weight and organ indexes of AIA rats. (A) weights of each group. (B,C,D) Spleen, liver and kidney indexes in each group (n=6). (E,F,G) Representative images of Spleens, livers and kidneys in each group. statistical significance was calculated by two-tailed Student’s t-test for two group comparison and one-way analysis of variance (ANOVA) for multiple comparisons. ###p<0.001 vs.Control group, *p<0.05 , **p<0.01 and ***p<0.001 vs Model group.

Table S1. The potential active components in serum of HT-treated rats

| **No** | **Name** | **Formula** | **Pubchem**  **id** | **Class** | **ppm** | **mzmed** | **rtmed** |
| --- | --- | --- | --- | --- | --- | --- | --- |
| POS_1 | Pyridoxine | C8H11NO3 | 1054 | Nicotinic acid alkaloids | 3.1 | 170.0807 | 63 |
| POS_2 | Norepinephrine | C8H11NO3 | 439260 | Tyrosine alkaloids | 3.1 | 170.0807 | 63 |
| POS_3 | 3-Hydroxy-2-methylpyridine | C6H7NO | 70719 | Nicotinic acid alkaloids | 3.9 | 110.0596 | 65.7 |
| POS_4 | 3-Pyridinemethanol | C6H7NO | 7510 | Nicotinic acid alkaloids | 3.9 | 110.0596 | 65.7 |
| POS_5 | Pyrogallol | C6H6O3 | 1057 | / | 3.3 | 127.0386 | 115.4 |
| POS_6 | Xanthosine | C10H12N4O6 | 64959 | Nucleosides | 1.7 | 285.0825 | 131.8 |
| POS_7 | Caprolactam | C6H11NO | 7768 | Lysine alkaloids | 3.6 | 114.091 | 217 |
| POS_8 | Fuziline | C24H39NO7 | 157773 | Pseudoalkaloids | 1.8 | 454.2791 | 222.6 |
| POS_9 | Hypaconine | C24H39NO8 | 76189740 | Pseudoalkaloids | 0.8 | 470.2745 | 222.8 |
| POS_10 | (E)-Osmundacetone | C10H10O3 | 9942292 | Phenylpropanoids | 4.1 | 161.059 | 273.4 |
| POS_11 | Ferulate | C10H10O4 | 445858 | Phenylpropanoids (C6-C3) | 4.2 | 177.0539 | 291.4 |
| POS_12 | 4-(3,4-dihydroxyphenyl)-7-methoxy-5-[(2S,3R,4S,5S,6R)-3,4,5-trihydroxy-6-(hydroxymethyl)tetrahydropyran-2-yl]oxy-chromen-2-one | C22H22O11 | 13962183 | Coumarins | 5 | 463.1212 | 298.2 |
| POS_13 | Umbelliferone | C9H6O3 | 5281426 | Coumarins | 4.5 | 163.0383 | 299.3 |
| POS_14 | 6-Hydroxycoumarin | C9H6O3 | 99477 | Coumarins | 4.5 | 163.0383 | 299.3 |
| POS_15 | Ononin | C22H22O9 | 442813 | Isoflavonoids | 4 | 431.1319 | 320.3 |
| POS_16 | 1-(4-methoxyphenyl)ethanone | C9H10O2 | 7476 | / | 4.6 | 151.0747 | 348.2 |
| POS_17 | Berlambine | C20H17NO5 | 11066 | Tyrosine alkaloids | 3.7 | 352.1167 | 357.1 |
| POS_18 | Thunberginol H | C17H16O5 | 13939150 | Coumarins | 3.8 | 283.0954 | 364.2 |
| POS_19 | Isoliquiritigenin | C15H12O4 | 638278 | Flavonoids | 4.1 | 257.0798 | 372.6 |
| POS_20 | 7-hydroxy-2-(4-methoxyphenyl)chromen-4-one | C16H12O4 | 5320693 | Flavonoids | 4.3 | 269.0797 | 380.9 |
| POS_21 | Isoformononetin | C16H12O4 | 3764 | Isoflavonoids | 4.3 | 269.0797 | 380.9 |
| POS_22 | 2-(2,6-dimethoxyphenyl)-5,6-dimethoxy-chromen-4-one | C19H18O6 | 629965 | Flavonoids | 4 | 343.1163 | 381.2 |
| POS_23 | 3,5,7-trimethoxy-2-(4-methoxyphenyl)chromen-4-one | C19H18O6 | 631095 | Flavonoids | 4 | 343.1163 | 381.2 |
| POS_24 | Isoxanthohumol | C21H22O5 | 513197 | Flavonoids | 4 | 355.1526 | 412.1 |
| POS_25 | (2S)-Isoxanthohumol | C21H22O5 | 9928523 | Flavonoids | 4 | 355.1526 | 412.1 |
| NEG_1 | 3-Hydroxybenzoic acid | C7H6O3 | 7420 | Phenolic acids (C6-C1) | 137.0241 | 254.7 | 1.9 |
| NEG_2 | Hydroxytyrosol | C8H10O3 | 82755 | Phenylethanoids | 153.0554 | 211.7 | 2 |
| NEG_3 | 2-Methoxycinnamic acid | C10H10O3 | 734154 | Phenylpropanoids | 177.0553 | 352.1 | 2.2 |
| NEG_4 | Salvianolic acid A | C26H22O10 | 5281793 | Lignans | 493.1129 | 305.2 | 2.3 |
| NEG_5 | [(2S,3R,4S,5S,6R)-3,4,5-trihydroxy-6-(hydroxymethyl)tetrahydropyran-2-yl] (2E,6E)-8-hydroxy-2,6-dimethyl-octa-2,6-dienoate | C16H26O8 | 14635445 | Monoterpenoids | 345.1548 | 269.7 | 2 |
| NEG_6 | 4-Hydroxycoumarin | C9H6O3 | 54682930 | Coumarins | 161.024 | 304 | 2.2 |
| NEG_7 | 3-(2,4-dihydroxyphenyl)-8-(1,1-dimethylallyl)-7-hydroxy-5-methoxy-chromen-2-one | C21H20O6 | 10090416 | Coumarins | 367.1179 | 405.5 | 2.1 |
| NEG_8 | N-[2-(4-hydroxyphenyl)ethyl]acetamide | C10H13NO2 | 121051 | Tyrosine alkaloids | 178.087 | 248.2 | 2.1 |
| NEG_9 | 1-(4-hydroxy-2-methoxy-phenyl)-3-(4-hydroxyphenyl)prop-2-en-1-one | C16H14O4 | 25201046 | Flavonoids | 269.0811 | 322.6 | 3 |
| NEG_10 | 4-Methoxycinnamic acid | C10H10O3 | 699414 | Phenylpropanoids (C6-C3) | 177.0553 | 352.1 | 2.2 |
| NEG_11 | (2Z,4E)-5-[(1R,3R,5S,8S)-3,8-dihydroxy-1,5-dimethyl-6-oxabicyclo[3.2.1]octan-8-yl]-3-methyl-penta-2,4-dienoic acid | C15H22O5 | 124680499 | Apocarotenoids | 281.1388 | 267.9 | 2.2 |
| NEG_12 | Griffonilide | C8H8O4 | 100341 | / | 167.0346 | 188.9 | 2 |
| NEG_13 | Okanin | C15H12O6 | 5281294 | Flavonoids | 287.0554 | 339.1 | 2.2 |
| NEG_14 | Rubiadin-1-methyl ether | C16H12O4 | 96191 | Polycyclic aromatic polyketides | 267.0656 | 383.3 | 2.4 |
| NEG_15 | 2-C-((Galloyloxy)methyl)-D-ribose 5-gallate | C20H20O14 | 21145076 | Phenolic acids | 483.0774 | 212.8 | 1.3 |
| NEG_16 | 4,5,5,15,15-pentamethyl-3,8,14-trioxatetracyclo[11.4.0.02,6.07,12]heptadeca-1(13),2(6),7(12),10,16-pentaen-9-one | C19H20O4 | 15108309 | Coumarins | 311.1282 | 466.3 | 2.1 |
| NEG_17 | 7-Hydroxy-4H-chromen-4-one | C9H6O3 | 5409279 | Chromanes | 161.024 | 304 | 2.2 |
| NEG_18 | 2'-Hydroxy-4'-methylacetophenone | C9H10O2 | 81338 | / | 149.0604 | 234 | 2.5 |
| NEG_19 | Coniferaldehyde | C10H10O3 | 5280536 | Phenylpropanoids | 177.0553 | 352.1 | 2.2 |
|  |  |  |  |  |  |  |  |

Table S2. The potential active components in serum of HT-treated rats

| **MS2.name** | **MS2**  **score** | **Formula** | **Class** | **KEGG pathway** | **P value** | **Q value** | **VIP** |
| --- | --- | --- | --- | --- | --- | --- | --- |
| Linoleyl-carnitine; AIF; CE0; CorrDec | 98.81 | C25H45NO4 | Fatty Acyls | NA | 0.00 | 0.00 | 2.06 |
| Docosahexaenoic acid | 98.21 | C22H32O2 | Fatty Acyls | map01040 | 0.00 | 0.00 | 2.10 |
| Cis-8,11,14-Eicosatrienoic acid | 98.12 | C20H34O2 | Fatty Acyls | map00591;map01040;map01100 | 0.00 | 0.00 | 1.50 |
| Cis-11,14-Eicosadienoic acid | 97.00 | C20H36O2 | Fatty Acyls | map01040 | 0.00 | 0.00 | 1.32 |
| FA 20:2 | 97.00 | C20H36O2 | Fatty Acyls | map01040 | 0.00 | 0.00 | 1.71 |
| Fingolimod | 97.00 | C19H33NO2 | Organonitrogen compounds | NA | 0.00 | 0.00 | 2.07 |
| 7Z, 10Z, 13Z, 16Z, 19Z-docosapentaenoic acid | 96.96 | C22H34O2 | Fatty Acyls | map01040 | 0.00 | 0.00 | 1.52 |
| cis-4,10,13,16-Docosatetraenoic Acid | 96.82 | C22H36O2 | Fatty Acyls | NA | 0.00 | 0.00 | 1.39 |
| 2-(3,4-dihydroxyphenyl)-5-hydroxy-10-isopropyl-9,10-dihydropyrano[2,3-f]chromene-4,8-dione | 96.41 | C21H18O7 | Flavonoids | NA | 0.00 | 0.01 | 1.18 |
| Docosatetraenoic acid | 96.18 | C22H36O2 | Fatty Acyls | map01040;map04216 | 0.00 | 0.00 | 1.60 |
| Octadeca-6,9,12,15-tetraenoic acid | 96.18 | C18H28O2 | Fatty Acyls | NA | 0.00 | 0.00 | 1.17 |
| LPA 18:2 | 96.03 | C21H39O7P | Glycerophospholipids | map00561;map00564;map01100;map01110;map04072;map04080;map04540;map04810;map04975;map04977;map05130;map05200 | 0.00 | 0.00 | 1.15 |
| Docosahexanoic acid | 94.74 | C22H32O2 | Fatty Acyls | map01040 | 0.00 | 0.00 | 1.50 |
| 1-Oleoyl-L-.alpha.-lysophosphatidic acid | 94.45 | C21H41O7P | Glycerophospholipids | NA | 0.00 | 0.00 | 1.31 |
| All-cis-4,7,10,13,16-docosapentaenoic acid | 93.60 | C22H34O2 | Fatty Acyls | NA | 0.00 | 0.00 | 1.41 |
| N-stearoyl taurine | 93.29 | C20H41NO4S | Fatty Acyls | NA | 0.00 | 0.00 | 1.16 |
| DL-Acetylcarnitine | 93.18 | C9H17NO4 | Fatty Acyls | NA | 0.00 | 0.00 | 1.80 |
| trans-Hexadec-2-enoyl carnitine | 91.62 | C23H43NO4 | Fatty Acyls | NA | 0.00 | 0.00 | 1.74 |
| 1-Palmitoyl Lysophosphatidic Acid | 89.32 | C19H39O7P | Glycerophospholipids | NA | 0.00 | 0.00 | 1.45 |
| Arachidonic acid | 89.11 | C20H32O2 | Fatty Acyls | map00590;map00591;map01040;map01100;map04216;map04217;map04270;map04611;map04664;map04666;map04723;map04726;map04730;map04745;map04750;map04912;map04913;map04921;map04923;map04925;map05140;map05146;map07034 | 0.00 | 0.00 | 1.75 |
| Eicosatetraenoic acid | 87.67 | C20H32O2 | Fatty Acyls | NA | 0.00 | 0.00 | 1.68 |
| 5,8,11,14-Icosatetraenoic Acid | 86.36 | C20H32O2 | Fatty Acyls | NA | 0.00 | 0.00 | 1.59 |
| 1-Hexadecanoyl-2-sn-glycero-3-phosphate | 85.91 | NA | NA | NA | 0.00 | 0.00 | 1.47 |
| 7-octadecynoic acid | 85.69 | C18H32O2 | Fatty Acyls | NA | 0.00 | 0.00 | 1.23 |
| FAHFA 20:4/20:3 | 84.71 | C40H64O4 | Fatty Acyls | NA | 0.00 | 0.00 | 2.32 |
| Octadeca-6,9,12-trienoic acid | 84.40 | C18H30O2 | Fatty Acyls | NA | 0.00 | 0.00 | 1.22 |
| 8-HETrE | 84.24 | C20H34O3 | Fatty Acyls | NA | 0.00 | 0.00 | 1.29 |
| Octadeca-8,10,12-trienoic acid | 84.20 | C18H30O2 | Fatty Acyls | NA | 0.00 | 0.00 | 1.21 |
| 3-Phosphoglyceric acid | 83.07 | C3H7O7P | Organooxygen compounds | NA | 0.00 | 0.00 | 1.04 |
| FAHFA 44:7 | 81.84 | C44H72O4 | Fatty Acyls | NA | 0.00 | 0.00 | 2.02 |
| FAHFA 42:7 | 80.21 | C42H68O4 | Fatty Acyls | NA | 0.00 | 0.00 | 1.42 |
| Cytidine | 79.28 | C9H13N3O5 | Pyrimidine nucleosides | map00240;map01100;map01232;map02010 | 0.00 | 0.01 | 1.91 |
| Gamma-linolenyl carnitine | 79.05 | C25H43NO4 | Fatty Acyls | NA | 0.00 | 0.00 | 2.58 |
| N-arachidonoyl taurine | 77.21 | C22H37NO4S | NA | NA | 0.00 | 0.00 | 1.68 |
| Cytidine-5'-monophosphate disodium salt,from Saccharomyces cerevisiae, crystalline | 72.19 | C9H14N3O8P | Pyrimidine nucleotides | map00240;map01100;map01232 | 0.00 | 0.00 | 1.50 |
| FAHFA 36:5 | 71.45 | C36H60O4 | Fatty Acyls | NA | 0.00 | 0.00 | 1.74 |
| 6,7-dimethoxy-8-oxo-8H-benzo[c]indolo[3,2,1-ij][1,5]naphthyridin-12-yl acetate | 71.29 | C22H16N2O5 | Indolonaphthyridine alkaloids | NA | 0.00 | 0.01 | 1.16 |
| N-Palmitoyl Glutamine | 71.24 | C21H40N2O4 | Fatty Acyls | NA | 0.00 | 0.00 | 1.35 |
| CPA(16:0/0:0) | 70.80 | C19H37O6P | Glycerophospholipids | NA | 0.00 | 0.00 | 1.14 |
| 4-amino-1-((2R,3R,4S,5R)-3,4-dihydroxy-5-(hydroxymethyl)tetrahydrofuran-2-yl)pyrimidin-2(1H)-one | 70.45 | C9H13N3O5 | Pyrimidine nucleosides | map00240;map01100;map01232;map02010 | 0.00 | 0.00 | 1.81 |
| U-75302 | 70.24 | C22H35NO3 | NA | NA | 0.00 | 0.00 | 2.00 |
| Xanthosine | 97.10 | C10H12N4O6 | Purine nucleosides | map00230;map00232;map01065;map01100;map01110;map01232;map02010 | 0.00 | 0.00 | 1.36 |
| Citric acid | 97.00 | C6H8O7 | Carboxylic acids and derivatives | map00020;map00250;map00630;map00720;map01053;map01060;map01061;map01062;map01063;map01064;map01065;map01066;map01070;map01100;map01110;map01120;map01200;map01210;map01230;map01240;map02020;map04742;map04922;map05230 | 0.00 | 0.00 | 1.74 |
| Lycodine | 97.00 | C16H22N2 | Phenanthrolines | NA | 0.00 | 0.00 | 1.10 |
| Bisphenol B | 97.00 | C16H18O2 | Benzene and substituted derivatives | NA | 0.00 | 0.00 | 1.04 |
| 2-Gonen | 97.00 | C17H26 | Steroids and steroid derivatives | NA | 0.00 | 0.00 | 2.00 |
| 1-Methyladenosine | 96.79 | C11H15N5O4 | Purine nucleosides | NA | 0.00 | 0.00 | 1.60 |
| Hydroxyphenyllactic acid | 95.13 | C9H10O4 | Phenylpropanoic acids | NA | 0.00 | 0.00 | 1.07 |
| N-lactoyl-phenylalanine | 93.36 | C12H15NO4 | NA | NA | 0.00 | 0.00 | 1.11 |
| L-a-Lysophosphatidylserine | 92.86 | C24H48NO9P | NA | NA | 0.00 | 0.00 | 1.36 |
| Dihydro-3-coumaric acid | 91.38 | C9H10O3 | Phenylpropanoic acids | map00360;map01100;map01120;map01220 | 0.00 | 0.00 | 1.21 |
| Betulin | 86.06 | C30H50O2 | Prenol lipids | NA | 0.00 | 0.00 | 1.04 |
| 11,17-Dihydroxy-3,20-dioxopregn-4-en-21-yl acetate | 85.56 | C23H32O6 | Steroids and steroid derivatives | NA | 0.00 | 0.00 | 1.42 |
| Imigliptin | 83.91 | C21H24N6O | Imidazopyridines | NA | 0.04 | 0.07 | 1.09 |
| Lyciumin D | 77.48 | C45H57N9O11 | Carboxylic acids and derivatives | NA | 0.00 | 0.00 | 1.98 |
| Tetranor 12-HETE | 77.21 | C16H26O3 | Fatty Acyls | NA | 0.00 | 0.00 | 1.63 |
| 4',5'-Dihydropsoralen | 74.91 | C11H8O3 | Coumarins and derivatives | NA | 0.00 | 0.00 | 1.21 |
| Isoplumbagin | 74.51 | C11H8O3 | Naphthalenes | NA | 0.00 | 0.00 | 1.78 |
| 3-Iodo-L-Tyrosine | 74.37 | C9H10INO3 | Carboxylic acids and derivatives | map00350;map01100;map04918 | 0.00 | 0.00 | 1.38 |
| LysoPI(18:1(9Z)/0:0) | 72.27 | C27H51O12P | Glycerophospholipids | map00564 | 0.00 | 0.00 | 1.71 |
| PI(20:4/0:0) | 72.10 | C29H48O12P1 | Glycerophospholipids | map00562;map00563-anchor biosynthesis);map00564;map00571 biosynthesis);map01100;map04070;map04136;map04138;map04140;map05132;map05152 | 0.00 | 0.00 | 1.20 |
| Anabasamine | 70.44 | C16H19N3 | Pyridines and derivatives | NA | 0.00 | 0.00 | 2.80 |
